# Associations between exposure to takeaway food outlets, takeaway food consumption, and body weight in Cambridgeshire, UK: population based, cross sectional study

**DOI:** 10.1136/bmj.g1464

**Published:** 2014-03-13

**Authors:** Thomas Burgoine, Nita G Forouhi, Simon J Griffin, Nicholas J Wareham, Pablo Monsivais

**Affiliations:** 1 UKCRC Centre for Diet and Activity Research, Medical Research Council (MRC) Epidemiology Unit, University of Cambridge School of Clinical Medicine, Box 285, Institute of Metabolic Science, Cambridge Biomedical Campus, Cambridge CB2 0QQ, UK; 2 Department of Public Health and Primary Care, Institute of Public Health, University of Cambridge, Cambridge, UK

## Abstract

**Objectives** To examine the association between environmental exposure to takeaway food outlets, takeaway food consumption, and body weight, while accounting for home, work place, and commuting route environments.

**Design** Population based, cross sectional study, using data on individual participants’ diet and weight, and objective metrics of food environment exposure.

**Participants** Working adults participating in the Fenland Study, Cambridgeshire, UK (n=5442, aged 29-62 years), who provided home and work addresses and commuting preferences. Takeaway food outlet exposure was derived using data from local authorities for individual environmental domains (at home, at work, and along commuting routes (the shortest route between home and work)), and for exposure in all three domains combined. Exposure was divided into quarters (Q); Q1 being the least exposed and Q4 being the most exposed.

**Main outcome measures** Self reported consumption of takeaway type food (g/day; pizza, burgers, fried foods, and chips) using food frequency questionnaires, measured body mass index, and cut-offs for body mass index as defined by the World Health Organization.

**Results** In multiple linear regression models, exposure to takeaway food outlets was positively associated with consumption of takeaway food. Among domains at home, at work, and along commuting routes, associations were strongest in work environments (Q4 *v* Q1; β coefficient=5.3 g/day, 95% confidence interval 1.6 to 8.7; P<0.05), with evidence of a dose-response effect. Associations between exposure in all three domains combined and consumption were greater in magnitude across quarters of exposure (Q4 *v* Q1; 5.7 g/day, 2.6 to 8.8; P<0.001), with evidence of a dose-response effect. Combined exposure was especially strongly associated with increased body mass index (Q4 *v* Q1; body mass index 1.21, 0.68 to 1.74; P<0.001) and odds of obesity (Q4 *v* Q1; odds ratio 1.80, 1.28 to 2.53; P<0.05). There was no evidence of effect modification by sex.

**Conclusions** Exposure to takeaway food outlets in home, work, and commuting environments combined was associated with marginally higher consumption of takeaway food, greater body mass index, and greater odds of obesity. Government strategies to promote healthier diets through planning restrictions for takeaway food could be most effective if focused around the workplace.

## Introduction

Neighbourhood food environments (“foodscapes”) have been labelled “obesogenic” when they facilitate the overconsumption of energy dense, nutrient poor foods, and increased levels of overweight and obesity.^1^ Understanding the influence of such foodscapes on diet and health has become more urgent with recent changes in society. During the past decade in the United Kingdom, consumption of food away from the home has increased by 29%,^2^ while the number of takeaway (or fast food) outlets has increased dramatically.^3^ These social and environmental trends could be contributing to rising levels of overweight and obesity.^4^ Policy makers are beginning to engage with the idea that unhealthy neighbourhood food environments might encourage unhealthy food choices,^5^ and could therefore be contributing to the obesity “epidemic.”^6^ As such, modifying the distribution and density of takeaway food outlets in cities and neighbourhoods is becoming an increasingly important element of nutrition and health policy in both the UK^7^ ^8^ ^9^ and United States.^10^ ^11^

Patronage of takeaway food outlets and overconsumption of takeaway foods have been linked strongly to low diet quality and to weight gain.^12^ ^13^ ^14^ ^15^ ^16^ ^17^ This link could be due to the types of foods obtained in these outlets, which tend to be energy dense,^18^ and because consumers often greatly underestimate their energy consumption when eating in these outlets.^19^ ^20^ However, despite increasing policy focus, identifying the association between exposures to unhealthy neighbourhood food outlets, diet, and body weight has proved challenging,^21^ ^22^ and the evidence base remains equivocal.^23^ ^24^ ^25^ ^26^ A recent systematic review found that, of those studies examining these exposures in relation to increased body weight, fewer than half reported positive associations.^25^ Even fewer studies have found positive associations with unhealthy dietary outcomes.^24^ ^25^ Overall, little of this research has been conducted in the UK.^27^ This evidence base is therefore not well placed to support governmental environmental interventions into the modification of supposedly obesogenic neighbourhoods.

Inconsistent findings among studies might be explained by differences in methods,^21^ including varying definitions of the term “neighbourhood,”^28^ differences in exposure metrics and food outlet type,^27^ and differences in assessment of dietary intake.^29^ However, many of these studies may be further limited by solely focusing on exposures to food outlets in residential neighbourhoods. There is a growing acknowledgment of “activity spaces,”^30^ the environments used by individuals to fulfil tasks and move between locations,^30^ which have been shown to extend well beyond the boundaries of residential neighbourhoods.^31^ ^32^ ^33^

Foods consumed away from the home are typically less healthy than those consumed at home.^18^ ^34^ Therefore, the environments around workplaces and commuting routes, for example, are important areas of study and potential targets for government policy intervention. One study found an inverse association between the density of takeaway food outlets in work neighbourhoods and body mass index, but no association with levels of exposure to takeaway food outlets surrounding the home.^35^ Other studies have focused on objectively captured daily estimates of food outlet exposure,^33^ or exposures to built and food environments along routes between home and non-home destinations.^36^ ^37^ ^38^ Substantial differences between takeaway food environments at home and away from home may underpin differential associations with dietary and weight outcomes in each setting.^33^ ^35^ ^39^ In summary, relying on estimates of takeaway food outlet exposure in residential areas alone is likely to underestimate total exposure within wider activity spaces, therefore compromising our ability to detect true associations between individuals and their environments.^39^

In a UK population based, cross sectional study with data on diet and measured body weight, we examined the extent to which exposure to takeaway food outlets in home and non-home environments was associated with consumption of energy dense takeaway foods, body mass index, and odds of overweight and obesity. We also tested for effect modification by sex.

## Methods

### Study sample

The Fenland Study is an ongoing, population based cohort study of adults aged 29-62 years (born between 1950 and 1975) in Cambridgeshire, UK (www.mrc-epid.cam.ac.uk/research/studies/fenland-study/), conducted by the MRC Epidemiology Unit. Recruitment began in 2005 from general practice lists in Ely, Wisbech, Cambridge, and surrounding villages. At the time of analysis, data for 10 452 participants were available. Participants completed questionnaires relating to their general lifestyle and medical history, and were weighed and measured by trained researchers.^40^ Participants also completed a food frequency questionnaire to assess the habitual consumption of foods.^41^ From the sample, we removed participants who had incomplete data for their work addresses (n=3129), lived or worked far outside Cambridgeshire (n=519), or did not report their travel mode to and from work (n=1729), including those who worked from home. These exclusions left a sample size of 5594. Despite these restrictions, our analytical sample remained representative of the full Fenland Study sample in terms of age, sex, body mass index, household income, and educational distributions (web table 1). All study procedures were approved by the Health Research Authority NRES Committee East of England-Cambridge Central.

### Exposure—food environment

We have previously described the methods for defining food environment exposures at home, at work, and along commuting routes.^39^ Briefly, participants’ home and work addresses were mapped by postcode using a geographic information system (ArcGIS 10, ESRI). Postcodes in the UK allow for relatively precise geocoding, with each postcode area containing only 15 addresses on average.^42^ Home and work neighbourhoods were defined as circular buffers with a 1 mile Euclidean (straight line) radius centred on these locations, based on previous studies suggesting that this definition relates closely to actual food purchasing behaviours among adults.^43^ Accurate data on food outlet locations^44^ ^45^ were sourced from 10 local councils covering the study area in December 2011, and again mapped by postcode. Food outlet listings are maintained by local councils to facilitate routine inspections of hygiene standards. 

All food outlets were classified by one researcher, based on a classification system for food environments developed by Lake and colleagues.^44^ Web table 2 provides details of our takeaway food outlet definition. UK local authorities typically categorise these outlets as “hot food takeaways” (class A5 under the Town and country Planning 2005 Use Classes Order).^9^ Planning restrictions are beginning to apply to these types of food outlets,^7^ ^8^ ^9^ making our definition of a takeaway food outlet particularly relevant to policy. We excluded workplace canteens and other food outlets not selling directly to the public from analyses. Numbers of takeaway food outlets and supermarkets were counted within participants’ neighbourhoods as a measure of outlet density; no denominator was necessary because of the standardised buffer size used to define neighbourhoods. Commuting routes were modelled according to the shortest distance along the street network between home and work addresses, using the ArcGIS Network Analyst and OS MasterMap Integrated Transport Network. 

Fenland Study participants also recorded their commuting travel modes and frequency. We allowed modelled routes and exposure to takeaway food outlets (counts) along these routes to vary by transport mode. We used 100 m route buffers for walking and cycling journeys; 500 m buffers for car journeys; and no buffers (assuming zero exposure) for bus journeys (except for walking buffers along the route from home to the nearest bus stop from home; and from the bus stop nearest to work, to work). For participants making multimodal journeys or for those using different modes of transport on different days, we calculated weighted route exposures, as described previously.^39^ ^46^

### Outcome—dietary intake, body mass index, and odds of overweight and obesity

A primary outcome variable was the consumption of specific energy dense foods that are commonly obtained from takeaway food outlets. Using data from the food frequency questionnaire, we estimated grams of daily intake of pizza, burgers, fried food (for example, fried chicken), and chips. Together, these foods provided a marker of takeaway type food consumption (g/day), referred to here as “takeaway food.” As a second primary outcome, body mass index was calculated from measured heights and weights. As a secondary outcome, we used World Health Organization cut-offs for body mass index to define under and normal weight (body mass index<25), overweight (≥25-30), and obesity (≥30).

### Statistical analyses

We used multiple linear regression models to estimate associations between takeaway food environments, and consumption of takeaway food and body mass index in the following environments:

- At home only
- At work only
- Along commuting routes only
- Exposures at home, at work, and along commuting routes combined.

We used multinomial logistic regression models to estimate associations when using overweight and obesity as outcomes. We modelled exposures to takeaway food outlets in all environmental domains as quarters of counts of takeaway food outlets (densities) using dummy variables (relative to the least exposed reference category, quarter 1). The addition of covariates into the models was theoretically informed a priori and included the following variables:

- Age, sex, total household income, and highest educational qualification (as proxies for individual level socioeconomic status)
- Car ownership (as a proxy for mobility beyond the immediate neighbourhood)
- Total energy intake (kJ/day, for models of takeaway food consumption only)
- Smoking status, for models of body mass index only
- Physical activity energy expenditure (kJ/kg per day), for models of body mass index only.

Physical activity was objectively assessed in the Fenland Study using combined heart rate sensors and accelerometers (Actiheart, CamNtech), which were worn for up to six days.^47^ ^48^ We calculated physical activity energy expenditure using the resultant intensity time series data,^49^ and added this as a continuous covariate into all models related to body weight. Participants with less than 48 h wear time (n=152) were excluded from analyses, resulting in an analytical sample for body mass index models of 5442 participants. We included education as a covariate because it was patterned by environmental exposure (web table 3), but we also conservatively adjusted for household income, despite a lack of obvious patterning. All models additionally controlled for the availability of supermarkets to account for food environment “context,” specifically to allow for the fact that takeaway type foods can also be purchased from supermarkets, and therefore to minimise confounding.^26^ Models accounting for journey exposure also adjusted for journey length, which could otherwise confound the association between commuting route exposure and outcomes of interest. We added interaction terms into our models, to test for the moderating effect of sex on our exposure-outcome associations.

In sensitivity analyses, we ran less conservative models that adjusted for each indicator of individual level socioeconomic status (education or household income) separately. We also conducted analyses omitting supermarket exposure as a covariate in statistical models. Finally, we ran models without adjusting for car access, because there was little heterogeneity in car access in this sample. All analyses were conducted using PASW Statistics 21.

## Results

### Sample characteristics

Table 1 shows descriptive statistics for the study sample. Men and women differed on several individual level characteristics, including energy intake, body mass index, and daily consumption of takeaway food. On average, this sample was exposed to 9.3 takeaway food outlets at home, 13.8 at work, and 9.3 along commuting routes. Individuals were therefore exposed to 48% more takeaway food outlet availability at work than at home. Mean exposure in home, work, and commuting route domains combined was 32.4 outlets.

**Table 1 tbl1:** Characteristics of participants in the Fenland Study sample, Cambridgeshire, UK

	Men (n=2470)	Women (n=2972)	All (n=5442)
Age	47.2 (7.4)	47.8 (7.1)	47.5 (7.2)
Energy (kJ/day)	8807 (2905)	7608 (2437)	8147 (2725)
Physical activity energy expenditure (kJ/kg per day)	59.9 (23.3)	49.4 (19.0)	54.2 (21.7)
Household income >£40 000 (no (%) of participants)	1389 (56.2)	1374 (46.2)	2763 (50.8)
Age at highest educational qualification >18 years (no (%) of participants)	883 (35.7)	886 (29.8)	1769 (32.5)
Current or ex-smoker (no (%) of participants)	1117 (45.2)	1278 (43.0)	2395 (44.0)
Owns car (no (%) of participants)	2321 (94.0)	2769 (93.2)	5090 (93.5)
Commuting travel mode (modal class)	Car	Car	Car
Anthropometric or dietary outcomes:			
Body mass index	27.1 (4.0)	26.3 (5.2)	26.7 (4.7)
Takeaway food consumption (g/day)	43.0 (33.0)	28.5 (25.9)	35.1 (30.2)
Food environment exposures*:			
Home supermarket availability	2.3 (3.1)	2.0 (2.8)	2.1 (3.0)
Home takeaway availability	9.9 (11.7)	8.9 (11.0)	9.3 (11.3)
Work supermarket availability	3.2 (3.5)	3.2 (3.2)	3.2 (3.4)
Work takeaway availability	13.4 (13.6)	14.1 (12.7)	13.8 (13.1)
Commuting supermarket availability	2.1 (2.4)	1.9 (2.2)	2.0 (2.3)
Commuting takeaway availability	9.6 (10.7)	9.0 (9.7)	9.3 (10.2)
Combined supermarket availability†	7.7 (5.9)	7.1 (5.6)	7.4 (5.7)
Combined takeaway availability†	32.8 (23.0)	32.0 (22.4)	32.4 (22.7)

Data are mean (standard deviation) unless stated otherwise. 4.18 kJ=1 kcal.

*Based on counts of food outlets across home, work, commuting route, and combined domains.

†Combined=home, work, and commuting route exposures combined.

### Associations between exposure to takeaway food outlets and intake of takeaway food

Exposure to takeaway food outlets was positively and significantly associated with consumption of takeaway food. Figure 1 plots β coefficients for the difference in consumption of takeaway food per quarter of exposure to takeaway food environments. These differences are shown according to home, work, commuting route, and combined environments. At home, individuals most exposed to takeaway food outlets (quarter 4) consumed significantly more takeaway food (β=4.9 g/day, 95% confidence interval 1.5 to 8.3, P<0.05) than those least exposed (quarter 1), but there was little evidence of a dose-response association between increasing exposure and consumption. 

**Figure fig1:**
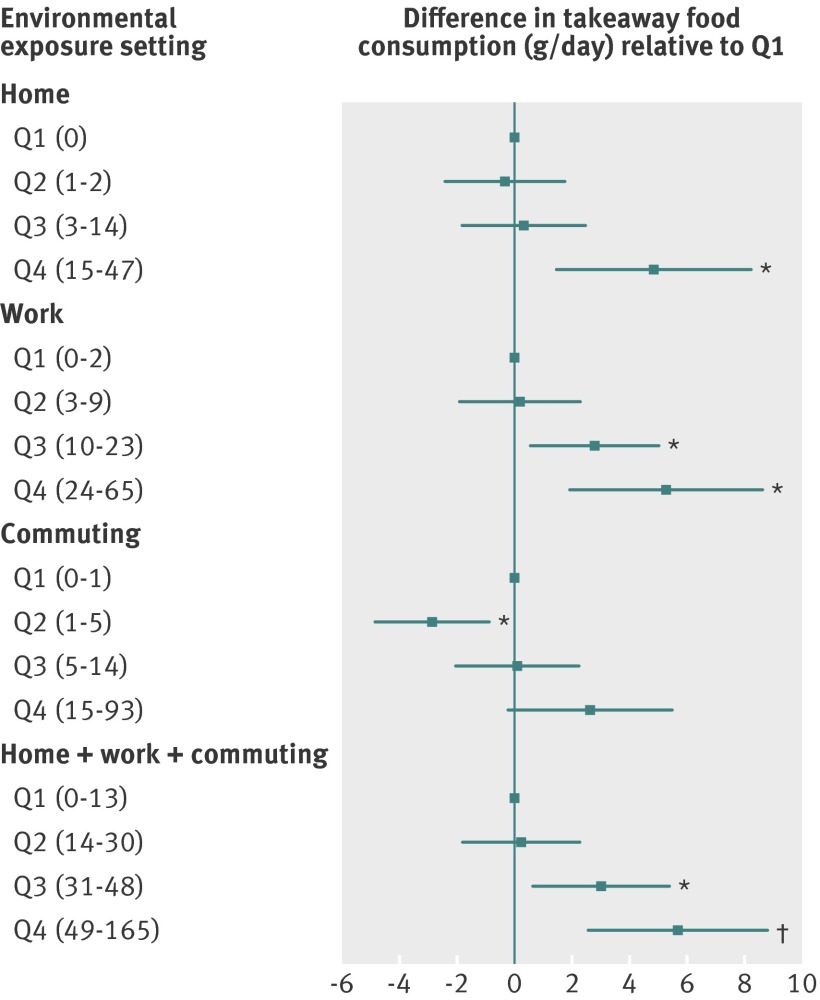
**Fig 1** Difference in consumption of takeaway food per quarter of exposure to takeaway food outlets, in the Fenland Study sample (n=5594). Differences are relative to the least exposed quarter (Q1). All models control for age, sex, household income, highest educational qualification, daily energy intake, car ownership, and supermarket availability. Commuting and combined models also adjust for journey length. *P<0.05. †P<0.001. Data points=β coefficients; error bars=95% confidence intervals; Q1=quarter least exposed to takeaway food outlets; Q4=quarter most exposed to takeaway food outlets. Numbers in brackets represent numerical limits (counts of food outlets) for each quarter of exposure

Exposure to food outlets in the work environment was positively associated with consumption of takeaway food, with evidence of a dose-response association. Individuals most exposed to takeaway food outlets at work consumed an additional 5.3 g/day (95% confidence interval 1.6 to 8.7) of takeaway food compared with those least exposed (P<0.05). For exposure along commuting routes, we saw little evidence of a trend in consumption across exposure quarters. In all environments combined, we saw evidence of a positive dose-response association with consumption of takeaway food. The most exposed quarter consumed an additional 5.7 g per day (2.6 to 8.8) compared with the least exposed quarter (P<0.001). Based on interaction terms, we found no evidence of effect modification by sex (P values across combined exposure quarters ranging from 0.173 to 0.761).

### Associations between exposure to takeaway food outlets and body mass index

Associations between exposure to takeaway food outlets and body mass index were equally consistent (fig 2). The group most exposed to takeaway food outlets (quarter 4) near home and along commuting routes, had a higher mean body mass index than those least exposed (difference of 0.97 and 0.65, respectively). However, there was little evidence of a dose-response association between exposures and outcomes in these settings. 

A positive dose-response association was observed between body mass index and exposure to takeaway food outlets near work. The most exposed group (quarter 4) had a higher mean body mass index (difference of 0.92, P<0.05), relative to those least exposed. In the model looking at combined exposures, we also saw evidence of a positive dose-response association between exposure and body mass index. The group most exposed to takeaway food outlets in all environments combined was estimated to have a body mass index 1.21 (95% confidence interval 0.68 to 1.74) greater than those least exposed (P<0.001). We found no evidence of effect modification by sex throughout (P values across quarters of combined exposure ranged from 0.292 to 0.705).

**Figure fig2:**
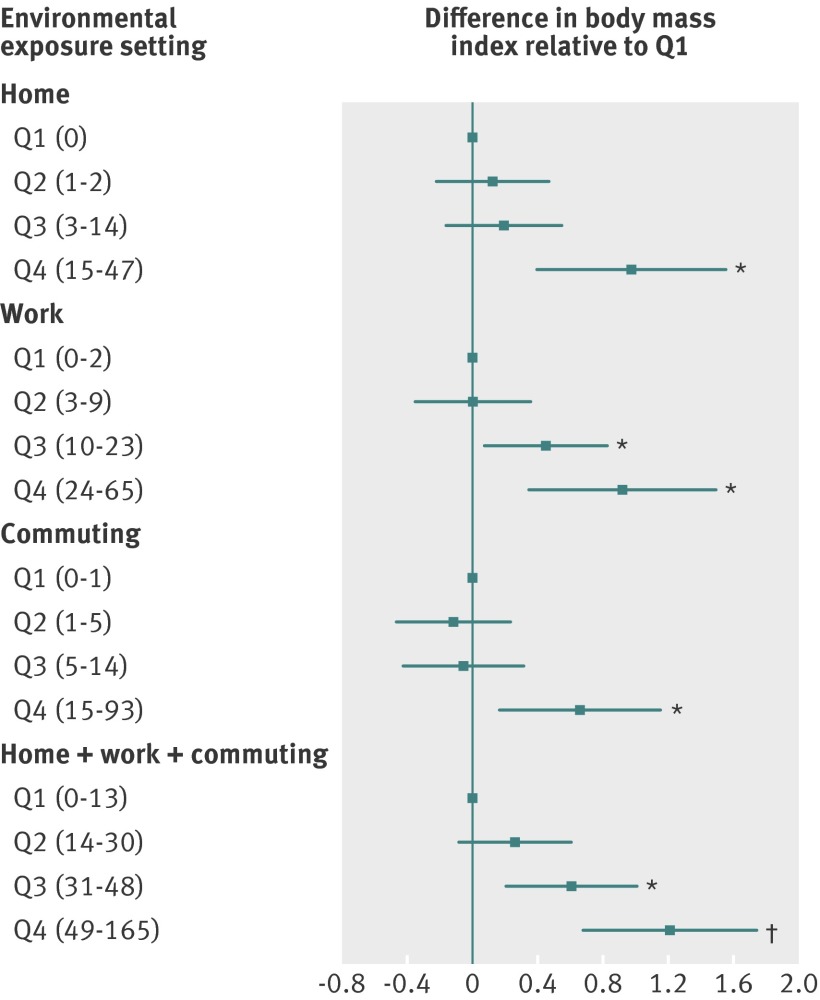
**Fig 2** Difference in body mass index per quarter of exposure to takeaway food outlets, in the Fenland Study sample (n=5442). Differences are relative to the least exposed quarter (Q1). All models control for age, sex, household income, and highest educational qualification, smoking status, physical activity energy expenditure, car ownership, and supermarket availability. Commuting and combined models also adjust for journey length. *P<0.05. †P<0.001. Data points=β coefficients; error bars=95% confidence intervals; Q1=quarter least exposed to takeaway food outlets; Q4=quarter most exposed to takeaway food outlets. Numbers in brackets represent numerical limits (counts of food outlets) for each quarter of exposure

### Associations between takeaway food outlet exposure and obesity

Table 2 shows results using multinomial logistic regression to model odds of overweight and obesity in relation to levels of takeaway food outlet exposure. Associations were more evident between environmental exposures and being obese than between exposures and being overweight. The group of people most exposed to takeaway food outlets (quarter 4) at home were more than twice as likely to be obese than be of normal weight (odds ratio 2.15, 95% confidence interval 1.50 to 3.10), relative to those least exposed (quarter 1; P<0.001). A dose-response association between odds of obesity and takeaway food outlet exposure along commuting routes was not apparent, but those most exposed showed 38% greater odds of being obese than those least exposed (1.38, 1.01 to 1.88). We observed a positive dose-response association in odds of obesity with respect to exposures at work; those most exposed showed a 47% greater odds of being obese compared to those least exposed (1.47, 1.03 to 2.10). Similarly, there was a dose-response association between the odds of obesity and exposure to takeaway food outlets in all domains combined; the group most exposed was more likely to be obese than those least exposed (1.80, 1.28 to 2.53).

**Table 2 tbl2:** Odds of being overweight and obese relative to being of normal weight, per quarter of exposure to takeaway food outlets, modelled using multinomial logistic regression analysis in the Fenland Study sample (n=5442), Cambridgeshire, UK

Environmental domain	Odds ratio (95% CI)	
	Odds of being overweight	Odds of being obese
At home		
Quarter 1	Reference	Reference
Quarter 2	1.03 (0.86 to 1.23)	1.06 (0.85 to 1.33)
Quarter 3	0.99 (0.82 to 1.190)	1.22 (0.96 to 1.54)
Quarter 4	1.26 (0.93 to 1.70)	2.15 (1.50 to 3.10)†
At work		
Quarter 1	Reference	Reference
Quarter 2	0.91 (0.76 to 1.09)	1.04 (0.83 to 1.30)
Quarter 3	0.90 (0.74 to 1.10)	1.28 (1.01 to 1.63)*
Quarter 4	0.95 (0.71 to 1.28)	1.47 (1.03 to 2.10)*
Along commuting route		
Quarter 1	Reference	Reference
Quarter 2	0.90 (0.76 to 1.07)	0.93 (0.75 to 1.17)
Quarter 3	0.92 (0.76 to 1.11)	0.97 (0.76 to 1.22)
Quarter 4	1.28 (0.99 to 1.65)	1.38 (1.01 to 1.88)*
All domains combined		
Quarter 1	Reference	Reference
Quarter 2	0.99 (0.83 to 1.19)	1.10 (0.88 to 1.38)
Quarter 3	1.09 (0.88 to 1.35)	1.32 (1.01 to 1.71)*
Quarter 4	1.27 (0.96 to 1.67)	1.80 (1.28 to 2.53)*

Odds ratios (95% confidence intervals) presented for being overweight and obese compared with being of normal weight, per quarter of exposure to takeaway food outlets relative to least exposed quarter (quarter 1). Body weight thresholds based on WHO standards: underweight and normal weight (body mass index <25), overweight (≥25-30), obese (≥30). All models control for age, sex, household income, and highest educational qualification, smoking status, physical activity energy expenditure, car ownership, and supermarket availability. Commuting and combined models also adjust for journey length.

*P<0.05.

†P<0.001.

### Sensitivity analyses

In models adjusting for only one measure of socioeconomic status at a time (household income or education), associations between exposure to takeaway food outlets and takeaway food consumption and body mass index were similar to the main analyses (web figs 1-4). Effect sizes throughout were generally greater when adjusting for household income only (web figs 1 and 2), although trends within and between exposure settings remained consistent. Excluding car access from our models had little effect on regression coefficients and their respective confidence intervals (results not shown).

In our analyses of takeaway food exposure, mutual adjustment for supermarkets proved to be critical. In models that omitted supermarket exposure as a covariate, the associations between combined takeaway food outlet exposure, consumption of takeaway food, and body mass index were attenuated towards the null (web figs 5 and 6, upper right panels, respectively). We also found an inverse association between combined supermarket exposure, takeaway food consumption, and body mass index (web figs 5 and 6, lower left panels, respectively). Again, we found that the associations were attenuated towards the null if mutual adjustment for exposure to takeaway food outlets was not made (web figs 5 and 6, lower right panels, respectively). 

## Discussion

To our knowledge, this is the first study of takeaway food outlet exposure to account for home, work, and commuting route environments, providing a more complete assessment of exposure to these outlets^50^ in relation to the consumption of energy dense takeaway foods and measured body mass index. We found evidence of an environmental contribution to the consumption of takeaway food and body mass index in all exposure domains studied. For exposure to takeaway food outlets in the work neighbourhood, we saw a strong and significant association between the density of takeaway food outlets, takeaway food consumption, and body mass index, with evidence of a dose-response effect. After combining the exposures for home, work, and commuting environments, we found a highly significant association between increased exposure to takeaway food outlets and consumption of takeaway food, body mass index, and odds of obesity. Again, the combined domains showed evidence of a dose-response effect, with especially large effect sizes for body mass index and odds of obesity.

In our analyses, compared with people least exposed to takeaway food outlets, we estimate those most exposed consumed an additional 5.7 g per day of takeaway food, which would constitute a 15% higher consumption than those least exposed. In a week, this translates into an additional 39.9g of takeaway food. This weekly amount constitutes more than half a small serving of McDonald’s French Fries (typically 71 g per serving)^51^ and about one quarter of the grams of takeaway food purchased per person per week in the UK in 2010.^52^

Other studies have found small but potentially meaningful effect sizes similar to those found in our study. A US based study found that a 1% increase in exposure to takeaway food outlets in the home environment was associated with a 0.13% increase in takeaway food consumption.^53^ From the current evidence base—which mostly focuses on the frequency of fast food consumption or fast food outlet visits in relation to body weight—it is difficult to determine whether this difference in consumption of takeaway food is clinically relevant in terms of its contribution to weight gain. However, longitudinal studies have found that increased patronage of fast food outlets was associated with excess weight gain over time in young adults.^13^ ^12^

It has also been suggested that studies generally find more robust environmental associations with body mass index than with diet.^54^ Indeed, associations were generally stronger with body mass index and odds of obesity than with diet in this study, although we found consistent and complementary associations between environmental exposure and both dietary and body weight outcomes throughout. Bias in our measurement of takeaway food consumption could explain why larger dietary effect sizes were not observed. Our measure was intended to be specific, although may not have been sensitive enough to capture all types of takeaway foods consumed. 

Omitting soft drink consumption from our analysis could also have underestimated the wider intake of takeaway food associated with exposure; we focused on energy dense takeaway foods in particular. People most exposed overall to takeaway food outlets in this study were estimated to have a body mass index more than one unit greater than those least exposed, and to have nearly twice the odds of being obese. These differences in body weight and odds of obesity are substantial, and might be cued by the increased consumption of takeaway foods, which we also observed. Unlike previous studies,^35^ ^53^ ^55^ we found no evidence to suggest that these associations varied according to sex. Trends in diet and body mass index were consistent between men and women in response to this environmental exposure.

Among the three foodscape domains (home, work, and commuting), we found the greatest environmental associations with consumption of takeaway food at work. This result suggests the notion that such consumption is both place and time specific,^39^ although more research is needed to investigate this hypothesis. A recent study identified an association between consumption of fast food at lunchtime and exposure to fast food outlets around school for children.^56^ Similarly at work, we suggest that time imperatives drive food purchases from more proximal food outlets, selling ready prepared meals,^57^ which could partly explain the strong associations observed in our study between these exposures and consumption of takeaway food.^58^

In relation to body mass index and odds of obesity, we found similar or stronger associations with maximum exposure to takeaway food outlets at home than with maximum exposure at work. However, this difference could indicate residual confounding, owing to unobserved environmental or social attributes covarying with exposure to takeaway food outlets in residential neighbourhoods only. Furthermore, workplace specific associations with body mass index have been found previously,^35^ and while this was not the case here, the dose-response nature of this association provides complementary evidence that this exposure domain could be important in the determination of body weight.

We observed the strongest and most significant environmental associations when combining the exposures at home, at work and along commuting routes, which is consistent with the notion that behaviours are best understood in the context of the activity space.^30^ The associations between exposure to takeaway food outlets, consumption of takeaway food, and body mass index were most consistent and robust, and were slightly stronger when domains were combined than in each domain separately. This association could reflect the greater and more realistic exposures estimated when accounting for the contributions of takeaway environments across three salient domains. Therefore, in developing our understanding of obesogenic environments, it is with caution that we should rely on studies suggesting a minimal environmental contribution to diet and weight from home neighbourhoods only.^33^ Such a limited appreciation of exposure could partly explain the limited and mixed evidence base that has developed around foodscape contributions to diet and adiposity.

Our models were also sensitive to the inclusion or omission of supermarket exposure as a covariate. This sensitivity reflects the importance of accounting for the wider food environment as opposed to assessing exposure to perhaps only one type of food outlet. Previous studies have called for the assessment of exposure to this wider collective environment,^26^ ^59^ ^60^ although such mutual adjustment is rarely made in the literature. Future studies should aim to provide a more comprehensive examination of food environment context, to detect more realistic associations between specific exposures and outcomes of interest.

### Implications for policy

Using local planning laws, policy initiatives have developed with the intention to limit neighbourhood access to sources of “unhealthy” food. These restrictions have historically been based on concerns over noise, litter, and neighbourhood aesthetics, but more recently have come to acknowledge the potential adverse effects of these food outlets on diet and health.^5^ ^61^ For example, in 2009, Waltham Forest Borough Council in the UK banned outlets selling hot takeaway food (class A5, similar to the types of food outlet examined here) within 400 m of schools, while using planning laws to limit clustering of hot food takeaways more generally throughout their London borough.^7^ The London Borough of Barking and Dagenham has similar restrictions on hot food takeaway outlets.^9^

Although these have been isolated initiatives so far, the principles of altering the geography of food availability to promote healthier choices have been most recently adopted by the *Takeaway Toolkit*, published by the Greater London Authority.^8^ This document encourages a more widespread uptake of this intervention where appropriate. However, it also notes that “these policies should be well thought through and evidence based.” As already stated, such policies are predicated on a limited understanding in the UK of the relationship between individuals and their environments. Our findings can therefore contribute to public policy, tentatively suggesting that such initiatives might help to reduce both takeaway consumption and body weight. Such interventions could be more effective if focused on work neighbourhoods, although environmental change in one setting may lead to behaviour change in another. For example, a person may adapt their commuting route to work in order to purchase takeaway food that is no longer available in the work neighbourhood. This represents a key question for future longitudinal studies.

### Methodological considerations and limitations

We used a 1 mile radius circular buffer to represent home and work neighbourhoods. However, neighbourhood definitions are likely to vary from person to person, or between home and work, and are influenced by subjective considerations (such as perceptions of safety, social cohesion, and “sense of place”^43^ ^62^). Our neighbourhood definition was theoretically linked to a distance that could be walked by an adult in 15 minutes,^63^ and based on a notion of neighbourhood extent described by participants in a study of UK adults.^43^ The limitations of using relatively arbitrary definitions of neighbourhood are also not unique to this study, and have been discussed previously.^39^ Although we accounted for food environment context to a degree, takeaway foods can be purchased from other types of food outlets (such as canteens at work, for which we had insufficient information). We did not control for the existence of these types of food outlets.

Our cross sectional study design prevented us from inferring causal associations between the environment, diet, and body mass index. Individuals with a preference for takeaway food consumption and increased body mass index may have simply opted for residential neighbourhoods with better access to takeaway food outlets. However, food consumption and body mass index were systematically associated with exposure at work, with self selection bias in the workplace probably less of a concern than at home. 

Temporal mismatch was also inevitable, which arises when data sources from different time points are used in cross sectional research.^59^ Because outcomes were measured in the Fenland Study between 2005 and 2013, and exposures were calculated using local council data from 2011, the consumption levels of takeaway food and body mass index values for some participants predated their estimated exposure level. This is a common consideration in research of this type.^21^ Moreover, we did not know how long our participants had been exposed to their current environments, both at home and at work. Recent residential moves and employment changes could render exposure to past foodscapes more important than to contemporaneous ones, thereby distorting our results.

As discussed previously,^39^ we acknowledge the potential inaccuracy of using the shortest street network distance to model commuting routes.^64^ ^65^ Furthermore, the accuracy of modelled commuting routes could vary by transport mode. Previous research suggests that the length of car journeys could be underestimated by shortest network routes, and the lengths of journeys on foot and by bike could be overestimated.^66^ However, increased journey length does not necessarily equate to greater exposure, so it is unclear how such inaccuracies might have biased our results.

The Fenland Study was designed to be representative of the Cambridgeshire region, achieving sample characteristics congruent with the region’s demographic characteristics (educated, employed, and white British). However, the sample may be less representative of other regions within the UK. Future research might consider focusing on the effects of food environment exposures on people of low socioeconomic status and unemployed people in particular, who may be more constrained to their residential neighbourhoods.^67^ Such focus was not possible here owing to the characteristics of the Fenland Study sample. Future research should also use longitudinal and experimental designs while accounting for residential and perhaps occupational histories. 

### Conclusions

This study used an innovative metric of exposure to takeaway food outlets, in multiple environmental domains, to explain patterns of takeaway food consumption and body mass index in a UK sample of working age adults. The study showed an environmental contribution to the consumption of takeaway foods, and especially to body mass index and the odds of being obese in this sample. In doing so, we provide novel UK evidence suggesting that policies designed to improve diets through restricting takeaway food availability may work, and may be most effective if focused around workplaces, where food availability of this type might be most important.

**What is already known on this topic**

In the UK, expenditure on foods consumed outside of the home has increased 29% in the past decade, accompanied by a proliferation of takeaways and other hot food outlets
Studies examining whether exposure to such food outlets can influence diet and body weight have mostly focused on residential neighbourhoods, with inconsistent findings
Despite the equivocal evidence, policies to promote healthier diets are increasingly based on the notion of reducing neighbourhood exposure to takeaway food outlets

**What this study adds**

We examined environmental exposure to takeaway food outlets, in Cambridgeshire, UK, based on domains at home, at work, and along commuting routes
Overall, access to takeaway food outlets in all three domains combined was positively associated with takeaway food consumption, body weight, and obesity
Policies designed to improve diets and bodyweight by restricting takeaway food access may work, and could be most successful if focused around the workplace

## Notes

We thank local councils for kindly supplying data to enable this work; all the volunteers who participated in the Fenland Study, as well as Fenland Study Coordination, Field Epidemiology, and data cleaning teams; and Søren Brage for his expertise on adjustment for physical activity energy expenditure.

Ethical approval: Fenland study volunteers gave written informed consent and the study was approved by the local research ethics committee for Cambridge. All other data analysed were in the public domain.

Data sharing: No additional data available.

Transparency: The lead author (the manuscript’s guarantor) affirms that the manuscript is an honest, accurate, and transparent account of the study being reported; that no important aspects of the study have been omitted; and that any discrepancies from the study as planned (and, if relevant, registered) have been explained.

## Notes

Cite this as: *BMJ* 2014;348:g1464
