# Supplementary material for: Associations between exposure to takeaway food outlets, takeaway food consumption, and body weight in Cambridgeshire, UK: population based, cross sectional study
Source: BMJ. 2014 Mar 13;348:g1464. doi: 10.1136/bmj.g1464 (PMC3953373; doi:10.1136/bmj.g1464)
Supplement: Supplementary file 1 [file burt015379.ww_default.pdf]

Web table 1: Fenland Study sample (n=10,452) and Fenland Study analytic sample (those with complete home/work, and transport preference data, n=5594) demographic comparisons.

	**Full Fenland Study Sample (n=10,452)**				**Fenland Study sub-sample (n=5594)**			
	Mean (SD)	Range	Median	IQR	Mean (SD)	Range	Median	IQR
Age	47.9 (7.4)	29.4 – 63.1	48.2	42.1 – 54.0	47.5 (7.2)	29.6 – 62.6	47.7	41.9 – 53.4
BMI	26.9 (4.8)	14.5 – 59.9	26.2	23.5 – 29.3	26.7 (4.7)	14.5 – 58.7	25.9	23.4 – 29.1
Sex, men (%)	46.1				45.4			
Household Income, >£40,000 (%)	49.8				50.8			
Education, >18yrs (%)	31.7				32.5			

Web table 2: Detailed takeaway food outlet characteristics, adapted from Lake and colleagues. [44]

**Takeaway food outlet**	**Characteristics**
Traditional	Hot food ordered and paid for at the till
	Wait whilst food is prepared and cooked
	No sit down option
	Usually open after 5pm
Traditional + delivery/collection option	The option to telephone for delivery and/or collection
With seating	Limited seating is available giving the option to eat-in
	No waiter service
With seating + delivery/collection option	Limited seating is available giving the option to eat-in
	No waiter service
	May have the option to telephone for delivery and/or collection

Web table 3: Descriptive statistics of sociodemographic characteristics, BMI and diet of adults in the Fenland Study analytic sample (n=5442)[a,b] across quartiles of combined takeaway food outlet density (at home, at work, and along commuting routes).

Combined Takeaway availability[c]	Male (%)	Age (yrs)	Household income, >£40,000 (%)	Education, >18 yrs (%)	Current or ex-smoker (%)	Owns car (%)	BMI (kg/m^2)	Energy intake[d] (kJ/day)	Physical Activity Energy Expenditure (kJ/kg/day)	Takeaway consumption (g/day)
1	45.1	47.7	51.2	25.3	41.1	97.4	26.7	8218	54.7	36.3
2	45.1	47.2	50.8	31.8	43.9	94.8	26.7	8138	54.1	34.6
3	44.7	47.3	50.9	32.2	44.3	93.2	26.6	8167	53.3	35.2
4	46.7	47.9	50.1	40.7	46.7	88.6	26.6	8084	54.4	34.2

[a] Means presented unless otherwise stated.
[b] Sample size reflects exclusions based on completeness of physical activity energy expenditure.
[c] 'Combined' Home + Work + Commuting route exposures.
[d] 4.18kJ = 1kcal.

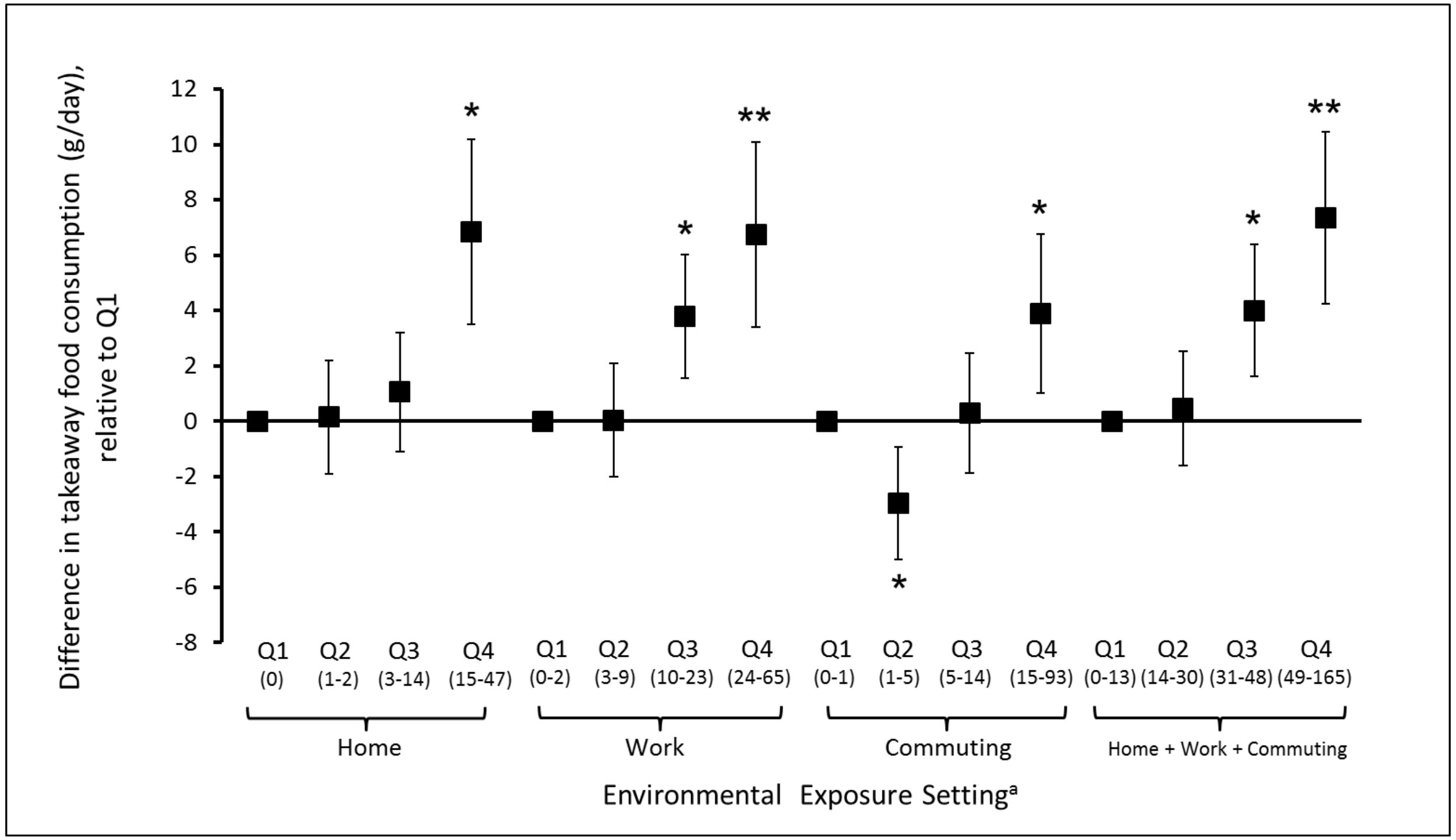

** p<0.001, * p<0.05.

[a] Q1, least exposed – Q4, most exposed to takeaway food outlets.

Web figure 1: Difference in takeaway food consumption (g/day) per quartile of takeaway food outlet density at home, at work, along commuting routes, and when combining home, work and commuting route exposures, relative to those least exposed (Q1), in the Fenland Study sample (n=5594), Cambridgeshire, UK. Data points represent β co-efficients, with error bars representing 95% confidence intervals. All models control for age, sex, household income *only*, daily energy intake (kJ/day), car ownership and supermarket availability. Commuting and combined models also adjust for journey length.

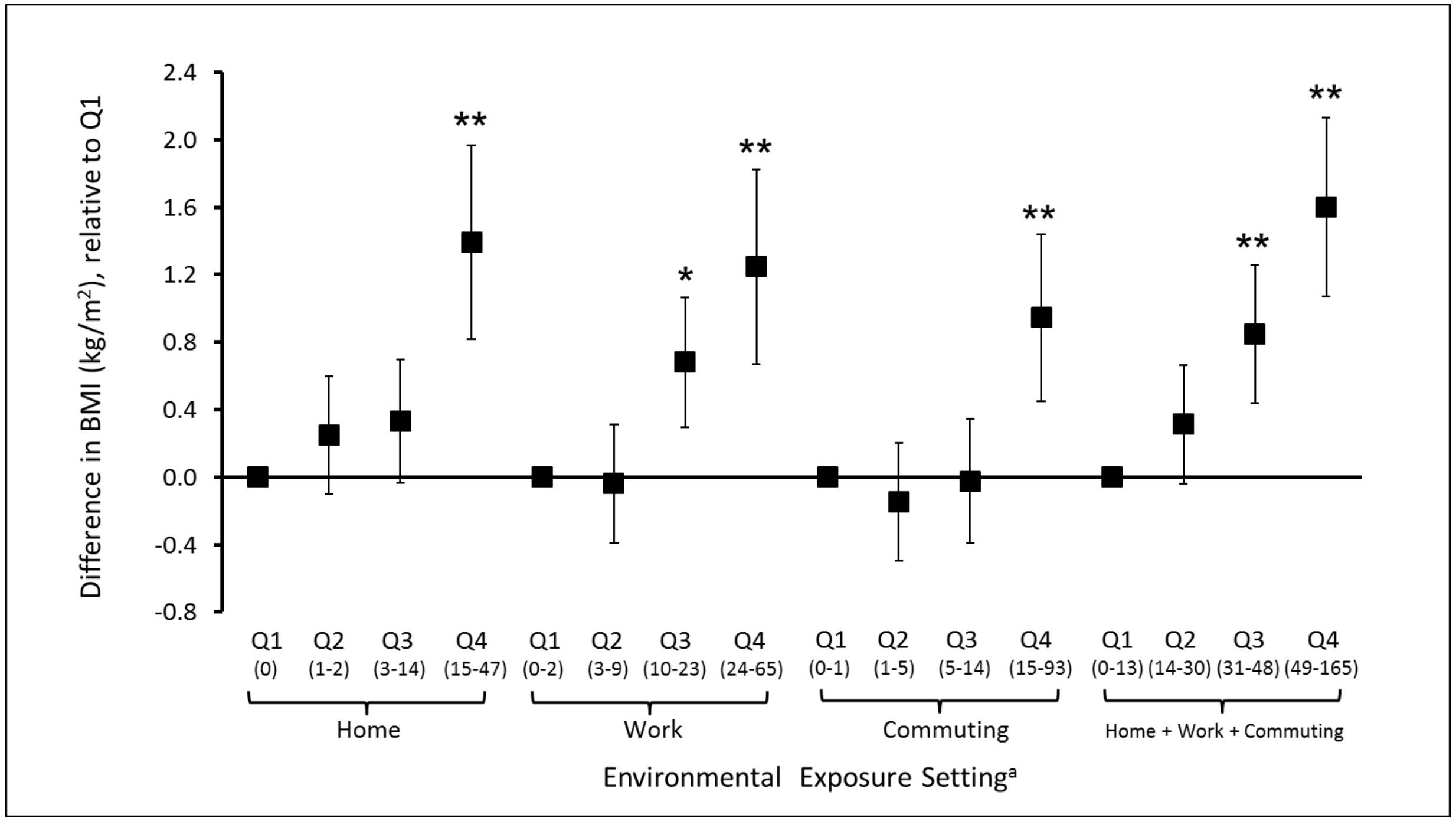

** p<0.001, * p<0.05.

[a] Q1, least exposed – Q4, most exposed to takeaway food outlets.

Web figure 2: Difference in body mass index (BMI) per quartile of takeaway food outlet density at home, at work, along commuting routes, and when combining home, work and commuting route exposures, relative to those least exposed (Q1), in the Fenland Study sample (n=5442), Cambridgeshire, UK. Data points represent β co-efficients, with error bars representing 95% confidence intervals. All models control for age, sex, household income *only*, smoking status, physical activity energy expenditure (PAEE, kJ/kg/day), car ownership and supermarket availability. Commuting and combined models also adjust for journey length.

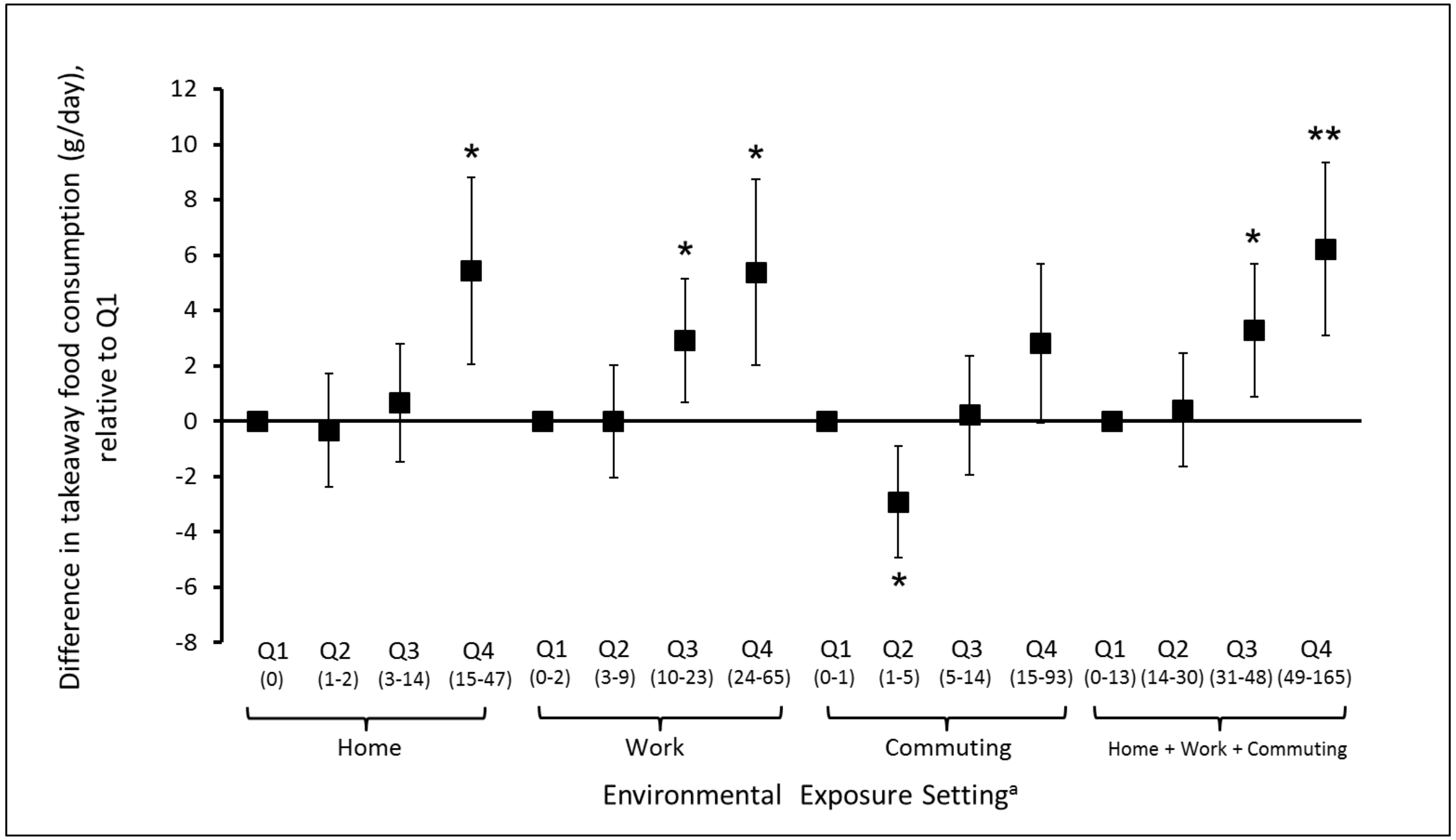

** p<0.001, * p<0.05.

[a] Q1, least exposed – Q4, most exposed to takeaway food outlets.

Web figure 3: Difference in takeaway food consumption (g/day) per quartile of takeaway food outlet density at home, at work, along commuting routes, and when combining home, work and commuting route exposures, relative to those least exposed (Q1), in the Fenland Study sample (n=5594), Cambridgeshire, UK. Data points represent β co-efficients, with error bars representing 95% confidence intervals. All models control for age, sex, education *only*, daily energy intake (kJ/day), car ownership and supermarket availability. Commuting and combined models also adjust for journey length.

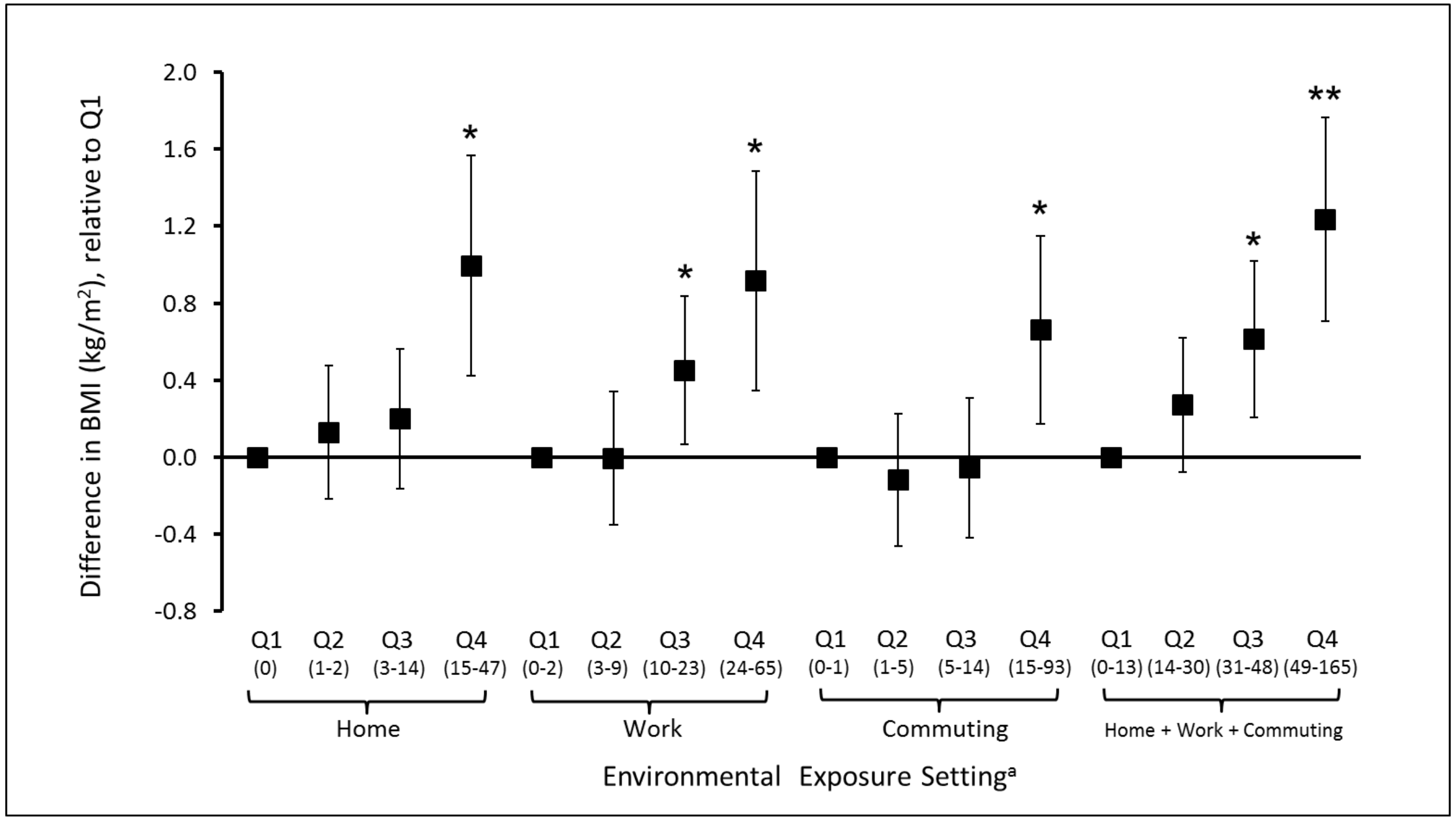

** p<0.001, * p<0.05.

[a] Q1, least exposed – Q4, most exposed to takeaway food outlets.

Web figure 4: Difference in body mass index (BMI) per quartile of takeaway food outlet density at home, at work, along commuting routes, and when combining home, work and commuting route exposures, relative to those least exposed (Q1), in the Fenland Study sample (n=5442), Cambridgeshire, UK. Data points represent β co-efficients, with error bars representing 95% confidence intervals. All models control for age, sex, education *only*, smoking status, physical activity energy expenditure (PAEE, kJ/kg/day), car ownership and supermarket availability. Commuting and combined models also adjust for journey length.

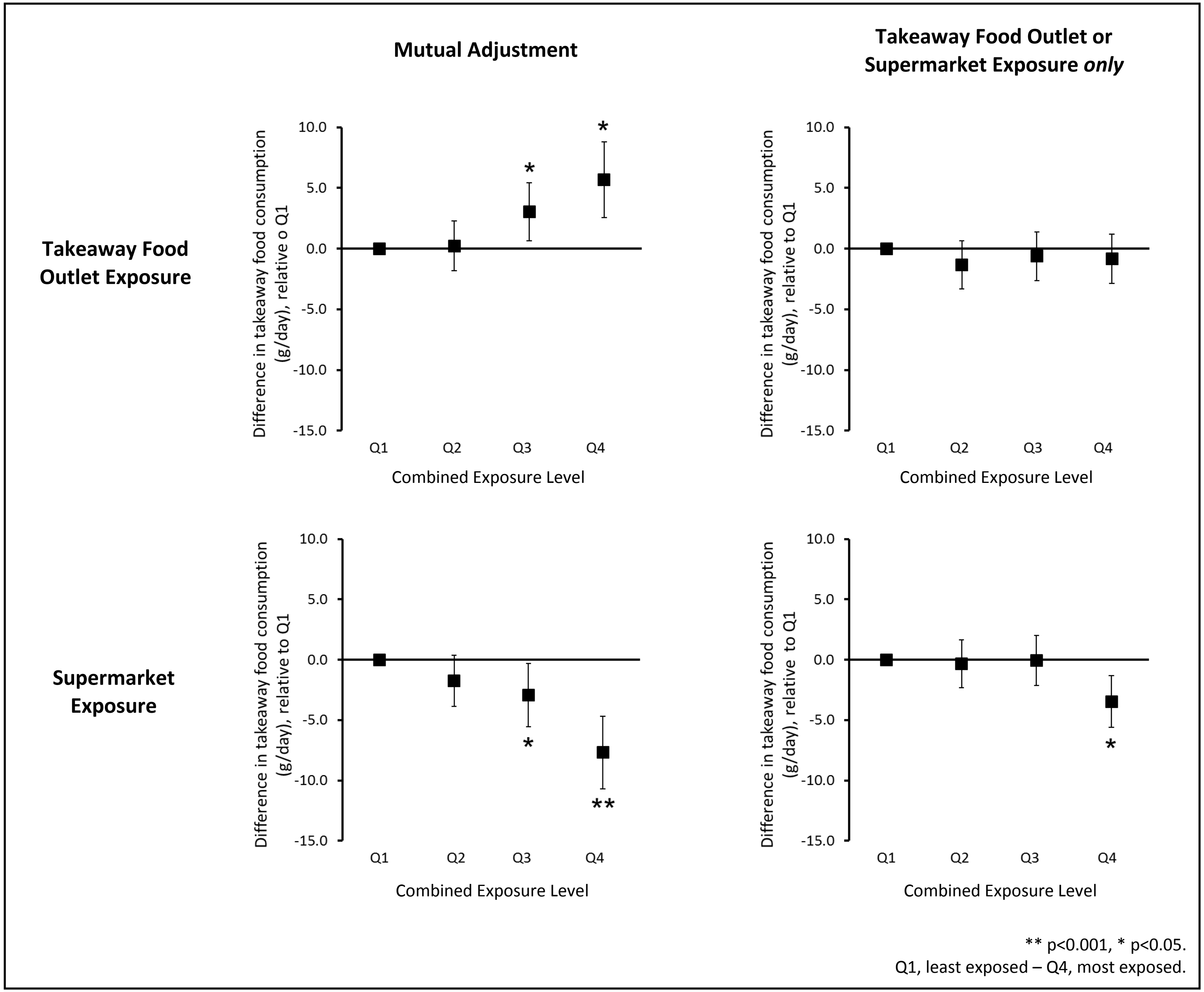

Mutual Adjustment
Takeaway Food Outlet or Supermarket Exposure *only*
Takeaway Food Outlet Exposure
Supermarket Exposure
Difference in takeaway food consumption (g/day), relative o Q1
Difference in takeaway food consumption (g/day), relative to Q1
10.0
5.0
0.0
-5.0
-10.0
-15.0
Q1
Q2
Q3
Q4
Combined Exposure Level
*
**
** p<0.001, * p<0.05.
Q1, least exposed – Q4, most exposed.

Web figure 5: Difference in takeaway food consumption (g/day) per quartile of takeaway food outlet or supermarket exposure (with or without mutual adjustment for takeaway food outlet or supermarket exposure) when combining home, work and commuting route exposures, relative to those least exposed (Q1), in the Fenland Study sample (n=5594), Cambridgeshire, UK. Data points represent β co-efficients, with error bars representing 95% confidence intervals. All models control for age, sex, household income, education, daily energy intake (kJ/day), car ownership and commuting route journey length.

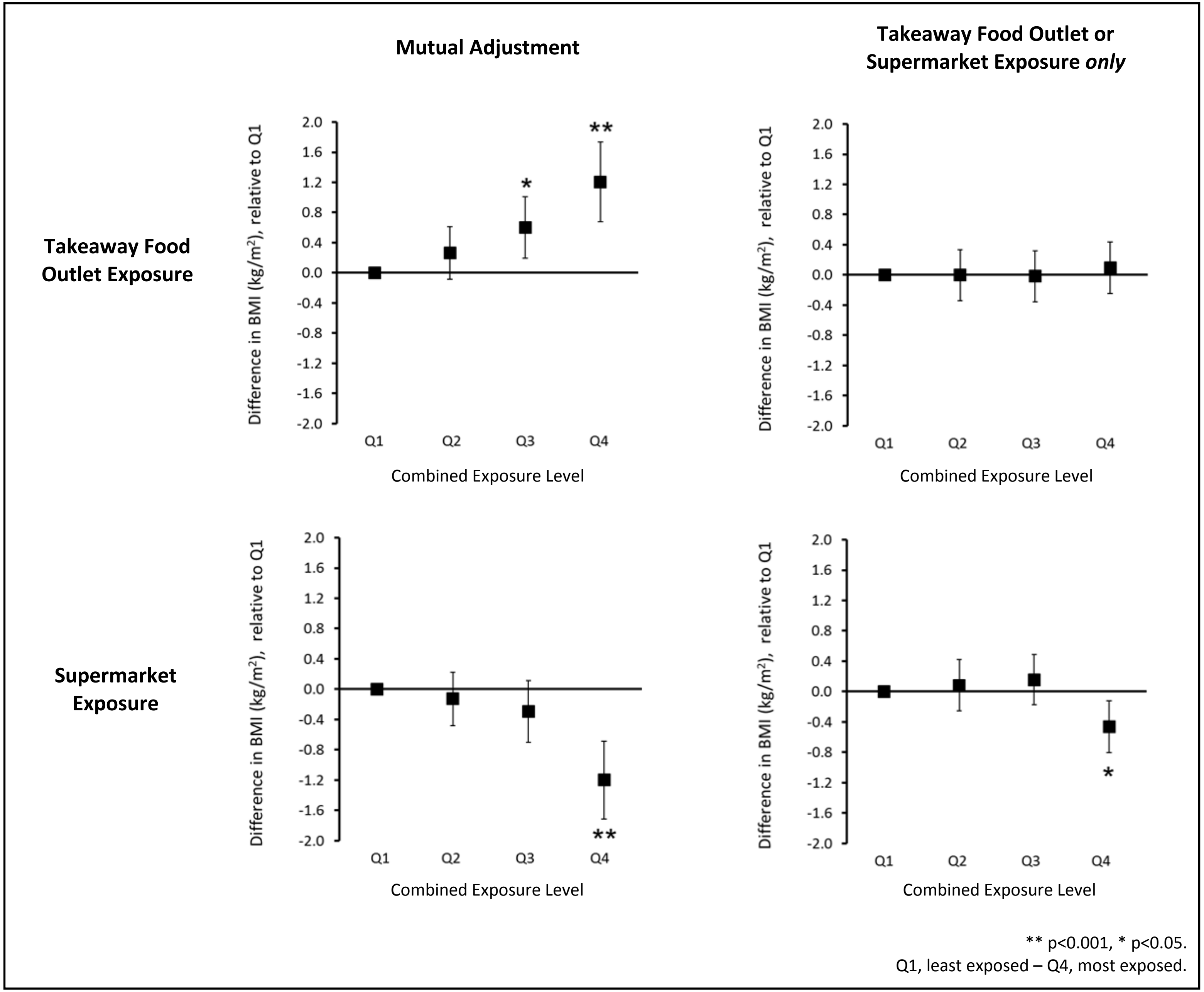
Mutual Adjustment
Takeaway Food Outlet or Supermarket Exposure *only*
Takeaway Food Outlet Exposure
Supermarket Exposure
Difference in BMI (kg/m²), relative to Q1
2.0
1.6
1.2
0.8
0.4
0.0
-0.4
-0.8
-1.2
-1.6
-2.0
Q1
Q2
Q3
Q4
Combined Exposure Level
**
*
** p<0.001, * p<0.05.
Q1, least exposed – Q4, most exposed.

Web figure 6: Difference in body mass index (BMI) per quartile of takeaway food outlet or supermarket exposure (with or without mutual adjustment for takeaway food outlet or supermarket exposure) when combining home, work and commuting route exposures, relative to those least exposed (Q1), in the Fenland Study sample (n=5442), Cambridgeshire, UK. Data points represent β co-efficients, with error bars representing 95% confidence intervals. All models control for age, sex, household income, education, smoking status, physical activity energy expenditure (PAEE, kJ/kg/day), car ownership and commuting route journey length.
